# The Future of Bridge Restorations

**Published:** 1921-02

**Authors:** Jas. Kendall Burgess


### THE FUTURE OF BRIDGE RESTORATIONS

BY JAS. KENDALL BURGESS, D.D.S.

The discussion of any future condition or relation implies contrast with the present. In order, therefore, that we may make an intelligent prophecy it seems expedient that we should discuss the present status of oral restoration and of its products. What are its virtues and what its vices as these matters concern the patient and the operator? What are the ideals being sought by its producers and how do these ideals comply with the fundamental requirements of the organism? What are its vital factors and what are some of the fads and fancies with which it is burdened? What is the relation between its cost and its value? These are matters of vital concern to both patient and operator, but more especially to the patient because it is the patient who pays in money, time, pain, nerves, apprehension and sometimes health and even life itself.

I had supposed that the term "bridge" was well and uniformly understood, but have been brought to believe that in the minds of some any type of partial denture is a bridge. For the purpose of this discussion, therefore, we shall divide partial restorations into two classes according to their means of support. Those depending upon the deep structure of the jaw, *i. e.*, the natural tooth or teeth with their root anchorages in the alveoli, for support and resistance to stress of mastication, we shall call bridges. Those dentures with their bases resting upon the superficial mucous tissues and depending upon such rest for support and resistance to the stress of mastication we shall call by their rightful names of saddle dentures. They are not bridges, by whatever means they may be steadied in their positions. To cement such an apparatus into position in the oral cavity would be criminal; to cement to position any appliance with such saddle-shaped base however supported is malpractice and, because some practitioners, ignorant or careless of the consequence of such procedure may do so, to place all workers in restoration who advocate fixed bridge work in the same category and the same condemnation as some seem prone to do is ignorant or slanderous according to the state of mentality of the accuser.

I have encountered, too, a very general misunderstanding of the term of "saddle," many dentists giving that name to any construction that approximates the gum tissue over an area larger than a mere point or broader than a single edge. The use of the word as it applies to oral restorations evidently comes from its similarity in conformation and its relation to the ridge to the saddle used by the rider of the horse or other beast of burden, by means of which the rider is seated and through which his weight is transferred to the animal that bears him. A cervical surface of a dummy tooth in a true bridge, which approximates in area a cross-section of that part of the natural tooth which it supplies and bears the relation of proximity to the tissues but does not gain its support from them, is not in any sense a saddle but merely a cervical surface.

We have restorations divided into two general classes: fixed and movable. We have removable restorations divided into two classes: bridge work and saddle dentures. We have saddle dentures with terminal anchorage and lateral anchorage and we have these with terminal anchorages divided into uni-terminal and bi-terminal anchorages.

### MOVABLE OR REMOVABLE

Removable bridge work of anything like general application is of such construction as to require certain parts which remain permanently fixed to the teeth; being fastened either externally, internally or occupying a combination of these relations. This combination of tooth and fixture constitutes the abutment to which must be attached by external, internal or a combination of these means some corresponding apparatus for the purpose of obtaining and maintaining an adequate security of anchorage for the restoration. This whole combination of fixture and detachable part or parts must be of such size and conformation as to come within the compass of the natural tooth or reasonably near to it, while at the same time each of the parts individually, whether fixed or detachable must be of substantial thickness and bulk and must occupy a corresponding amount of space.

It is clearly to be seen then that if we consider the sum of the spaces necessary for each and all of these parts in its relation to the total bulk of the tooth, by the time sufficient tissue has been sacrificed for their placement the tooth will be but a wreck of its former self—pulp gone, enamel gone, in some cases the entire crown gone, the roots reamed to a shadow and nature's beautiful handiwork but a dream and a memory to be replaced with cunningly devised barbarities of the manufacturing jeweler.

In considering the relative cost to the patient there must be charged against removable bridge work the following items:

Greatly multiplied pulp removals in the field of operation—practically universal in some systems—and greatly increased destruction of the hard tissues of the teeth extended frequently to include the entire crown of the tooth;

Largely increased expenditure of time for this multiplication of exacting detail;

Anxiety or even mental distress to many patients due to natural revulsion to dismemberment and fear of the consequences physically and esthetically;

Corresponding expenditure of nervous energy consequent upon apprerension as well as upon physical pain and discomfort;

Increased financial outlay;

The risk always and in some cases the materialization of periapical and systemic infection through the destruction of pulp in teeth used as abutments;

Hazard and sometimes the realization of puncture of roots with consequent lessening of their usefulness or even their loss through excessive enlargement for the placement of retentive devices;

The peril and often the actuality of fracture and loss of abutments as the ultimate result of the same procedure;

The danger, often fulfilled, of discoloration and consequent loss of esthetic harmony from the same cause;

Annoyance at the necessity of constant removal for cleaning and in many cases acute embarrassment at the perpetual reminder of physical impairment.

Notwithstanding this exorbitant difference in the cost to the patient, when we come to compare the relative values to the patient of these restorations it will be found, as we will show in discussing the matter from the scientific aspect, that there is no service or function of bridge work, active, prophylactic or esthetic, performed by the removable bridge that is not performed equally as well or better by the fixed bridge; so that the removable bridge has no advantage to offer in the character, quality or longevity of its service to compensate for its disadvantages.

It seems to me a misguided policy to condemn fixed bridge work promiscuously because some, even much, of it is poorly planned and made, in favor of removable bridge work when removable bridge advocates are continually at loggerheads among themselves, the father and followers of each system casting anathemas at the progenitors and promulgators of each other's systems for what each claims to be the failure of the other's efforts and the shortcomings of their systems. Since all engaged in the general work of restoration must of necessity do removable denture work, the essayist being no exception,

I protest that as strong as is my aversion to this unnatural procedure, the fact of its being removable is my smallest objection, but the violent perversions of nature, the destruction of her organs and tissues and functions in the name of restoration are revolting to humane instincts, let alone the finer sensibilities. What a grim and hollow mockery to murder and mutilate and then bedeck the carcass with the exquisite trappings of the jeweler's art!

### THE OPERATOR

Just as the financial may be the smallest item in the patient's expense account, so the money cost of his productions may be the least significant item to the operator. Some of the larger items are beyond the reach of money. I have used the term "outlay" to include and describe everything the operator puts into the securing of his results.

The complicated parts and devices necessary in generally accepted types and systems of removable bridge work, and the surgical care of the teeth and pulp canals necessitated by their use, demand:

A degree of skill not possessed and incapable of development by many operators and expensive in time, money and effort for its development by those whose innate ability make it possible;

The care of infinite, complicated and exacting details not only for their production but for their adjustment and correlation requiring an expensive amount of time and a corresponding expenditure of physical and nervous energy;

Delicate, complicated and expensive instruments and apparatus and sometimes manufactured parts;

The necessity in many cases of accepting fees disproportionate to all this outlay in time, skill, energy and money;

Increased risk of failure complete or partial both as regards the physical and esthetic value of the abutment teeth and the attendant ill-will of the patient and loss of prestige.

To make all of this worth while or justifiable, the value of the results obviously must be commensurate with the expenditure on the part of both patient and operator, and as the results must be measured by the comparative benefit to the patient of the two methods under immediate consideration it will be necessary to study briefly the functions of restorations and make our comparisons in the light of these findings. This will bring us to a consideration of our subject from the scientific point of view.

Every restorative operation to fulfill its complete mission, mechanical, prophylactic and esthetic, must comprise good surgery, good engineering, good art and good workmanship. Aside from the surgical procedure necessary to eradicate morbid conditions and to place the field of operation in a physiological condition as a preliminary to any kind of restoration, perhaps the most important function of surgery is prophylaxis, and prophylaxis worth the paper it takes to write the word on must conserve and protect against destruction through operative violence as well as through degenerative processes.

The removal of pulp and tooth tissue for the accommodation of removable bridge parts and devices not only belies the conservative phase of prophylaxis, but renders the tissues not destroyed by this mutilating procedure more vulnerable and more susceptible to degenerative influences, and through the periapical connecting link between these tissues and the vascular system places every organ and tissue dependent upon the vascular supply in jeopardy through its contamination.

Compare with this pulp and tissue waste, this disturbance of function and risk to the organism, the simple surgical procedure necessary for the preparation of abutment teeth for modern fixed bridge work where tissue removal is scarcely as extensive or as deep as that for ordinary cavity preparation, and it is plainly evident that the advantage surgically is entirely on the side of fixed bridge work.

It is no less true when we consider the matter as an engineering problem. The mechanical function of mastication is a matter of engineering, and so far as it depends upon the establishment of occlusal relations, there is no reason why any advantage should lie with either type. But the constant removal of the detachable part of removable bridge work necessitates a grasp of such increased tenacity of the fixed parts to the abutment teeth to guard against dislodgement as is not generally secured, or to be secured in teeth anterior to the molars except by anchorage into the root canals; thus transforming a pillar or column effect of the entire tooth with its ability to bear external stress without injury into a tube of greatly lessened capacity to stand the strain which now becomes an interior one. And not only so, but the anchoring attachments are of such complications and consequently of such size as to require an amount of space which can only be gained by the removal of so much and of such a proportion of the interior tissues of the root as that the amount and the proportion remaining about this enlarged tubular interior is left so thin as to be greatly weakened and its liability to fracture and consequent loss correspondingly increased. Such a procedure, by tearing down and weakening the foundation and undermining the superstructure, violates the most elementary principles of engineering which should maintain the integrity of the foundation in the greatest possible degree and take every advantage of its inherent strength.

As an artistic undertaking, where the method of construction necessitates the removal of the natural crown and the use of an artificial substitute, the patient, in frequent instances, is subjected to a distinct loss in cosmetic harmony and excellence and the discoloration of the natural tooth following pulp removal is to many patients an esthetic calamity. Comparison of the almost universal destruction of the pulp in teeth used as abutments for removable bridge work with its infrequency in modern fixed bridge prosthesis will show the fixed bridge to have the advantage where art is concerned, not only in measure as these blemishes occur, but in the proportion in which the patient is subjected to the risk of their occurrence.

### THE TECHNIQUE

The technical details and requirements of removable bridge construction are so numerous and so exacting and its difficulties so many and so great that a proportionately smaller number of men are capable of mastering them, whereas those of fixed-bridge construction are so elemental in comparison that any man capable of practicing dentistry at all should be able to overcome them. The method or system whose technical requirements bring it within the capabilities of the largest possible number of would-be producers certainly is advantageous in the degree in which its simplicity makes accurate production possible. So here again we must look to the modern fixed bridge for a way out of the mechanical intricacies and difficulties into which the mistaken ideals of the removable bridge enthusiasts have led us.

### HYGIENIC BRIDGE WORK

Under this head must be considered also hygienic prophylaxis because the details of construction that make for or against this important feature are in the hands and under the responsibility of the workman. It is refreshing to read in the published articles and books of the removable bridge propagandists that one of the chief advantages of removable bridge work over fixed bridge work is the fact that it can be taken out of the mouth and cleaned.

If an object allowed to remain in the oral cavity will collect matter which is or becomes unsanitary, and if this unsanitary condition is inimical to the patient's welfare, it requires but elementary reasoning to determine that the greater the extent of surface exposed in the oral cavity the greater will be the amount of accumulation and the element of danger is correspondingly increased.

I have seen enough to convince me that patients, with rare exceptions, are extremely careless about the condition in which they keep any type of restoration that can be taken out and handled and replaced, and that the most serious attempts at sanitary hygiene are in mouths where teeth remain as nature intended they should and where the patient, realizing that they must be cleansed in the mouth if at all, will put forth the effort to do it.

Oral sanitation is a personal equation, but oral surgical prophylaxis or conservation is a professional duty and obligation. I have no sympathy with the maudlin demand for a degree of oral cleanliness, amounting to sterilization, beyond nature's plans and requirements, and with all the straining after it beyond possibility of attainment while in the effort to achieve it nature's plans and purposes are thrown to the winds in the sacrifice of her tooth pulps and crowns and the uncovering of her blood stream to the danger of contamination.

The fixed bridge of modern construction where the dummy teeth are individualized presents three surfaces, the lingual, the buccal and the morsal of the same conformation, in the same alignment and bearing the same relation to the cleansing apparatus. Its mesial and distal surfaces being united over a part of their areas present reduced surfaces minus any contact points for cleansing. Its only addition to the five surfaces of the natural teeth is the gingival surface, reduced in area, approximating the gum, but not bearing upon it, admitting no large particles of debris, but allowing the free passage of floss silk and rinsing fluids, and is amenable to sanitary care in the mouth.

A careful consideration of every phase of the scientific requirements of oral restoration fails to reveal any point of superiority of the removable bridge over the modern fixed bridge to compensate for its obvious sacrifices, perils and difficulties.

### ECONOMIC PHASE OF ORAL RESTORATION

The facts established concerning the various items of cost to patient and producer in our consideration of the patient's point of view and the producer's point of view bears directly on the economic question as it affects these two individuals as parties to the transaction. It is to be assumed that having entered into the transaction each of these parties is conversant with these facts and items and is prepared to fulfill his obligation with reference to them. I do not know the cost in time and money to the producer or to the patient of the various systems of removable bridge work, but it is inconceivable that it can compare in economy from this point of view with modern fixed bridge work.

It must be patent to the most casual observer that there is a great economic waste going on in the dental profession, and while no doubt it will always be so, in some measure it will be increasingly so as long as men capable of large production of a service which meets the genuine needs of the many are guilty of this extravagant waste of time and energy in the practice of Butchery de Luxe and oral jewelry for the few. This seems to me to make out an invulnerable case for modern fixed bridge work *vs.* removable bridge work.

When we come to compare that class of saddle dentures having biterminal fastenings, for which fixed bridge work might be substituted on the one hand or removable bridge work on the other, we find them to occupy a middle ground. Gaining their support from the superficial tissues they require in many instances less security of anchorage to the adjacent teeth than is required for removable bridge work. This admits of simpler and less destructive devices than those for removable bridge work, but necessitates more complicated ones than those required for fixed bridge work. These matters weigh directly for or against the patient and producer as the case may be. And so we might continue to sum up the advantages and disadvantages through the whole list of viewpoints, and it will be discovered that where there is a difference in values the superiority as between the saddle denture and the removable bridge will be on the side of the saddle denture, and as between the saddle denture and the fixed bridge it will rest with the fixed bridge.

The next study of relative values comes naturally between the individual saddle with the biterminal anchorage and the saddle with lateral anchorage obtained by means of the cross bar for the upper jaw and the lingual bar for the lower jaw. It is obvious that the greater the number of available points of anchorage the wider the distribution of the stress and the less the stress that comes on any one point. Where, therefore, this lateral security can be added to the terminal points of anchorage the strain on these terminal points is reduced. This in many instances will admit of greater conservation of the tissues of the individual teeth used for anchorage purposes and is proportionately better surgery even though it does necessitate an additional tooth at the remote end of the bar that must furnish anchorage and that may need some alteration for that purpose.

The increased number of anchorage points certainly enhances the value of this restoration as an engineering project, especially in cases where there is a weakened anchorage of one or more of the abutments. Its relative value esthetically need not be less and may be greater, depending upon the type of anchorage used at the terminals. The chief point to be urged against it in this comparison is that the bar presents a limited surface and thickness of foreign material to which the patient must become accustomed. The gain in surgical and engineering value, however, outweighs in many cases this small inconvenience and gives the advantage to the saddle and bar denture. This is especially true where restorations are required for both sides of a jaw and where a bilateral saddle denture, obtaining mutual support for both sides from the bar anchorage, can be used instead of two individual biterminal saddle dentures.

When we come to make a comparison of this laterally anchored saddle, or bar and saddle denture, with removable bridge work, if we agree that it is to be preferred to the biterminal denture and that this biterminal denture is a more satisfactory measure than the removable bridge, we naturally conclude that the bar and saddle denture is a superior restoration to the type of removable bridge under discussion. And that this is true can not be refuted on a scientific basis. It is an incalculable gain in surgery, engineering and workmanship. Its relative value esthetically depends upon the method of utilizing the abutment or anchorage teeth.

### COMPARATIVE MERITS OF THE FIXED BRIDGE AND THE BAR AND SADDLE DENTURE

Coming to the final comparison where there is opportunity for a choice—*i. e.*, between the fixed bridge of modern construction and the bar and saddle denture—it will be found that where a type of attachment is used for the bar and saddle composed of two parts, one of which must be and remain fixed to the anchorage tooth, there is no gain surgically over the simple preparation necessary for the bridge and that where clasps are used what is gained in surgery is lost in art on account of the display of metal. The advantage from the engineering standpoint may rest with either type according to the conditions and relations that obtain in the individual case. The details and difficulties of workmanship are more numerous and greater in the case of the bar denture. The fixed bridge has all the advantages to the patient of nearness to nature in conformation and fixation. It costs the producer less in time and money to construct, and while there is not the same disparity economically between the fixed bridge and the saddle and bar denture as there is between the fixed bridge and the removable bridge, there is a worth-while difference in favor of the fixed bridge.

It is not possible or necessary to make fair comparisons between dentures that *may be* fixed and those that *must be* removable, or between two types of removable dentures where inherent conditions eliminate the use of one of them. But there is a class of case which presents an opportunity for choice between two types of saddle dentures. This is the case where the molars are lost.

The simple anchorage used for the biterminal saddle denture will not suffice for the available single end and additional anchorage must therefore be secured. This brings a choice in many cases between destruction of the pulps in the bicuspids for purposes of compound anchorage at the single terminal or the use of lateral anchorage for the saddle by means of the cross bar for the upper jaw and the lingual bar for the lower. With two possible exceptions comparison from every point of view favors the lateral anchorage of the bar denture. First, it may or may not be less desirable from the artistic point of view according to the method of terminal anchorage pursued, and, second, it presents the bar already described to which the patient must become accustomed. But in comparison with the atrocity of pulp and tooth destruction generally pursued for the terminally anchored individual saddle and the travesty on engineering which the whole project comprises, the impediment of the bar sinks into insignificance. This is increasingly true in cases where the need of bilateral restoration admits of the use of two saddles mutually supported by the bar instead of the two individual terminally anchored saddles.

### THE CLASP BRIDGE

The sponsors for the clasp bridge have never claimed any but the most limited application for that production.

This being true, it can scarcely be compared fairly with those types or systems of either removable or fixed bridge work of general application. Where it can be used it gains immeasurably over the class of removable bridge work I have described on the surgical and engineering sides and requires less destruction of tissue even than the fixed bridge; but its slight gain surgically over the fixed bridge is outweighed by its inferiority from the engineering, the esthetic and the workmanship points of view. The technique of its construction is more exacting and more difficult than is that of the fixed bridge and it has the disadvantage to the patient of never becoming an integral part of the organism.

### THE ORAL RESTORATION OF THE FUTURE

So much for the present status of oral restoration and the result of the efforts being made to fulfill its mission. I do not believe the confusion in which we find it is confined to this particular branch of our calling. I believe I am safe in saying that there never was a time in the history of the profession when the whole of its theory and practice was in such complete disorder, with confusion in every department, disagreement on every point and with no definitely settled plan of action for anything pertaining to it. Our thought is not fixed on any one method of procedure; no principle seems to be universally established; no operation standardized. The specialists in each of its departments are separated into schools and factions and the general practitioners running hither and thither after this champion of an idea or that promulgator of a system.

In the matter of oral restoration, as in all other things, we stand between the past and the future, between history and prophecy, and while it has not seemed necessary to delve into the dark ages, I have tried briefly to outline the conditions that have come down to us through evolution as the heritage of that history with such faults and virtues as it possesses, not only as a basis for prophecy, but as an incentive to the correction of its evils and the more intelligent guidance of its progress and control of its destinies.

What the future will bring in the matter of the relation of oral pathological conditions to systemic disturbances, and what will be its bearing on oral restoration, must be a mere matter of quantity and not of quality. It may mean more or fewer restorations. None can tell, but I have no reason to believe it will vary greatly. At the moment we "laymen" bid fair to be ground to bits between the upper and the nether millstones of the pessimists and the optimists regarding focal infections.

The pendulum swings first to this side and then to that, but I think it will come to a stop presently over a sane middle ground. Our investigators will learn to classify conditions and relations as we do not know how to do now, and when they do we will extract some teeth that we are trying to save now and save some under conditions where we extract them now and the average will probably be maintained.

But whether we extract more or fewer teeth will not alter the character of the restorations made necessary nor the principles upon which they should be constructed. So that it is an easy and safe prophecy that there will always be plenty of restorative work to do.

But what of the restorations that must supply the need? Necessarily they must be either fixed or removable. Our awakened moral consciousness will force men to forego the petty considerations that influence them to this system or to that method and establish their efforts upon nature's own fundamental requirements: surgery, engineering, art and workmanship. With this bettering of the professional conscience and the enlightenment of our clientele now rapidly progressing, the ill-fitting, gingival, irritating shell-crown atrocity and the slipshod kind of bridge construction that usually goes with it will be discarded along with that cut-throat and blood-sucking class of oral mechanical contrivances that regards nothing of God's creation sacred enough to stand in its ruthless path, whose perpetrators put prophylaxis to the sword and place sanitation on its throne, and the sum total of whose contribution to the advancement of scientific restoration has been to take it out of the blacksmith's shop and put it in the jeweler's laboratory.

There will be more scientific and a better variety of attachments and refinements of construction for the saddle denture to be used when the engineering possibilities will not admit of fixation, and some means also, not so set about with difficulties and exactions of technique but that the man with moderate skill can use it. There will always be places and no doubt some demand for limited specialties like the clasp bridge, but I am of the opinion that its technical difficulties will keep it less in favor than the virtues of the well-constructed specimens deserve.

But when the basic principles of oral restoration, surgery, engineering, art and workmanship, are understood and accepted, and the simplicity and accuracy of their application to the construction of modern near-to-nature fixed bridge work put into effect, I am fully persuaded that this will be the accepted type of restoration where conditions, from the engineering point of view, will admit of a denture without saddles for its support. The small amount of tissue destruction it necessitates, its pulp conservation and prophylactic value commend it surgically; its grasp upon the foundation teeth and its mechanical efficiency comprise good engineering; the inconsequential display of metal in its attachments gives it esthetic worth; and the ease of its construction bring it within the capabilities of any man of average ability.

Its cost in time and money to both patient and producer makes it accessible to those who can pay for any but the crudest restorations.

There is nothing else in bridge or plate prosthesis that approaches it in nearness to nature. What more can be said?—Extracts from an article in *The Dental Cosmos* for December, 1920.

